# Supplementary figures and images for: Photosynthesis and Salt Exclusion Are Key Physiological Processes Contributing to Salt Tolerance of Canola (Brassica napus L.): Evidence from Physiology and Transcriptome Analysis
Source: Genes (Basel). 2022 Dec 20;14(1):3. doi: 10.3390/genes14010003 (PMC9858917; doi:10.3390/genes14010003)

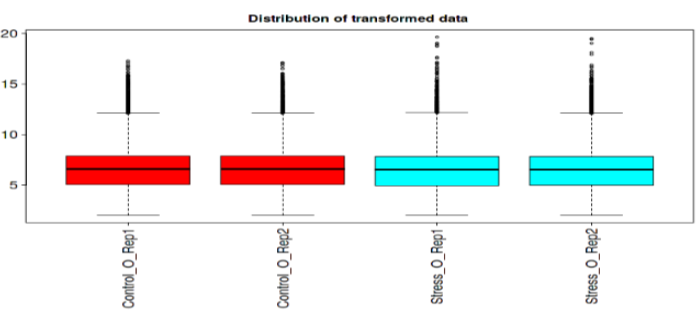

Supplement: Supplementary file 1 [file genes-14-00003-s001.zip › Figure S1.tif]
